# Heterologous Prime–Boost Vaccination with GRA35-Encoding DNA and mRNA Vaccines Enhances Protective Immunity Against *Toxoplasma gondii* Infection in Mouse Models

**DOI:** 10.3390/microorganisms14051000

**Published:** 2026-04-29

**Authors:** Sisi Chen, Rui Li, Yanyan Zhu, Jie Sun, Jia Chen

**Affiliations:** 1 Department of Neurosurgery, Ningbo Key Laboratory of Nervous System and Brain Function, The First Affiliated Hospital of Ningbo University, Ningbo 315010, China; 2 Health Science Center, Ningbo University, Ningbo 315211, China

**Keywords:** *Toxoplasma gondii*, vaccine platform, nucleic acid vaccines, prime–boost strategy, lipid nanoparticles, protective immunity

## Abstract

*Toxoplasma gondii* is an obligate intracellular protozoan parasite that causes toxoplasmosis, posing a significant threat to human health and livestock production worldwide. Although monovalent DNA or mRNA vaccines often confer only partial protection, whether these platforms can be effectively integrated into a heterologous prime–boost regimen against *T. gondii* remains to be fully elucidated. Here, we constructed GRA35-encoding DNA and mRNA vaccines and evaluated their immunogenicity and protective efficacy, administered either alone or in heterologous prime–boost combinations, in C57BL/6 and BALB/c mice. Both vaccines induced strong antigen-specific immune responses, with the heterologous prime–boost regimen eliciting the strongest effects and conferring the most robust and consistent protection across both mouse strains. Immunization triggered a predominantly Th1-skewed response characterized by significantly elevated IFN-γ production, accompanied by balanced antigen-specific IgG responses. Moreover, vaccinated mice developed rapid and potent cytotoxic T lymphocyte (CTL) responses. Following challenge with the RH and PRU strains, vaccinated mice exhibited prolonged survival and significantly reduced brain cyst burdens following PRU challenge compared with control groups. Collectively, these findings indicate that GRA35-based nucleic acid vaccines, particularly when administered in a heterologous prime–boost regimen, elicit multifaceted protective immune responses and represent promising vaccine candidates against *T. gondii* infection.

## 1. Introduction

*Toxoplasma gondii* is an obligate intracellular protozoan parasite infecting approximately one-third of the global population and causing toxoplasmosis [1]. While infection is typically asymptomatic in immunocompetent individuals, it can result in severe disease in immunocompromised patients and congenital infection with serious neurological consequences [2]. In addition, toxoplasmosis poses a substantial threat to livestock, particularly small ruminants, leading to abortion and neonatal mortality and significant economic losses [3]. These factors underscore the need for effective and safe vaccination strategies to control both human and veterinary toxoplasmosis [4].

Nucleic acid vaccines have emerged as promising platforms for parasitic disease control. DNA vaccines targeting *T. gondii* have been extensively investigated, and numerous antigen candidates including surface antigens, dense granule proteins, and rhoptry effectors have demonstrated varying degrees of protective efficacy in murine models [5,6,7]. More recently, mRNA vaccines have attracted increasing attention due to their favorable safety profile, rapid production capability, and strong induction of both cellular and humoral immunity [8]. Although only a limited number of studies have evaluated mRNA vaccines against *T. gondii*, preliminary findings suggest that this platform may provide effective immune activation. Nevertheless, monovalent DNA or mRNA vaccination alone often induces partial protection, and strategies to enhance immunogenicity remain necessary [9,10]. Importantly, these limitations suggest that antigen selection alone may not be sufficient to achieve robust protection, highlighting the need for optimization of vaccine platforms and delivery strategies.

Heterologous prime–boost strategies, which sequentially combine distinct vaccine platforms, have been shown to enhance immune responses in several infectious disease models by leveraging complementary mechanisms of antigen expression and innate immune activation [11,12,13]. In *T. gondii*, heterologous regimens such as DNA prime-protein boost have demonstrated improved immune responses compared with single-modality vaccination [14,15]. In other pathogens, combinations involving mRNA vaccines and viral vectors or DNA platforms have been reported to amplify both antibody and T-cell responses [16,17]. However, whether DNA and mRNA vaccines can be effectively integrated into a heterologous prime–boost regimen against *T. gondii*, and whether such combinations provide advantages over homologous strategies, remains unclear.

Dense granule proteins (GRAs) are secreted effectors that play critical roles in parasite invasion, intracellular survival, and modulation of host immune responses, making them attractive vaccine candidates [18]. Among these, GRA35 has been identified as an important virulence-associated factor, and our previous work indicates that a GRA35-based DNA vaccine is capable of inducing antigen-specific immune responses and partial protective immunity against *T. gondii* infection in mice [19]. Building upon this foundation, the present study was designed not to re-evaluate the immunogenicity of GRA35 per se, but to use GRA35 as a model antigen to investigate how different vaccine platforms and immunization strategies influence the quality and magnitude of the immune response. Specifically, this study sought to determine whether mRNA-based vaccination can enhance the immunogenicity of GRA35, and whether heterologous prime–boost regimens combining DNA and mRNA platforms can further improve protective efficacy. To address these questions, the present study aimed to construct GRA35-encoding DNA and mRNA vaccines and to systematically assess their immunogenicity and protective efficacy, administered either alone or in heterologous prime–boost combinations, in both C57BL/6 and BALB/c murine models of acute and chronic toxoplasmosis. This study sought to evaluate whether heterologous DNA–mRNA prime–boost immunization could enhance both humoral and cellular immune responses, thereby improving protection against acute and chronic T. gondii infection across different mouse strains. Ultimately, this work aimed to explore the potential of cross-platform nucleic acid vaccination as a strategy to optimize protective immunity against intracellular parasitic infections.

## 2. Materials and Methods

### 2.1. Mice

C57BL/6 and BALB/c female mice (6–8 weeks of age) raised under specific pathogen-free conditions were obtained from the Zhejiang Experimental Animal Center (China) and have been established as suitable models for *T. gondii* vaccine evaluation in prior investigations [20]. All animal procedures were reviewed and approved by the Animal Research Ethics Committee of Ningbo University (approval no. SYXK(ZHE)2019-0005, 3 April 2023).

### 2.2. Parasites, Antigens, and Cells

Tachyzoites of the *T. gondii* ME49 strain and RH strain were maintained and propagated in human foreskin fibroblast (HFF) cell culture as previously described [21]. Tachyzoites of the ME49 strain were utilized for GRA35 production, while the RH strain tachyzoites and PRU strain cysts were employed for mouse challenge experiments. Cysts of the *T. gondii* PRU strain were propagated and maintained in specific pathogen-free mice and used for chronic infection following our previously established protocol [21]. *Toxoplasma* lysate antigen (TLA) was prepared from freshly isolated tachyzoites collected from the peritoneal cavity and purified as described previously [22]. HEK 293T and HFF cells were cultured at 37 °C in a humidified atmosphere containing 5% CO^2^ using Dulbecco’s modified Eagle’s medium (DMEM; Gibco, NY, USA) supplemented with 10% fetal bovine serum (FBS; Gibco, USA), 100 U/mL penicillin, and 100 μg/mL streptomycin.

### 2.3. Construction of Recombinant pVAX1-HA-GRA35 Plasmid

Total RNA was extracted from *T. gondii* (ME49 strain) tachyzoites and subjected to reverse transcription–PCR (RT–PCR). For construction of the eukaryotic expression plasmid pVAX1-HA-GRA35, the full-length coding sequence of GRA35 (Gene ID: 7897592) was amplified by PCR using cDNA, which was synthesized and used as a template for amplification. The amplified fragment was generated using the following primers: GRA35F (5′-TAGCGCTAGCGCCACCTACTTTGCCCGACTACTG-3′) and GRA35R (5′-ATCGCTGCAGGAATGATTTTGGAGCGGGCCAC-3′). The PCR product was digested with NheI and PstI and ligated into the corresponding restriction sites of the pVAX1-HA vector to generate an in-frame fusion with the HA tag.

The recombinant plasmid was transformed into *Escherichia coli* DH5α competent cells and purified using an endotoxin-free plasmid extraction kit (Macherey-Nagel, Düren, Germany) according to the manufacturer’s instructions. Purified plasmids were re-suspended in sterile PBS and stored at −20 °C until use. DNA concentration and purity were assessed by spectrophotometric measurement of absorbance at 260 nm and 280 nm.

### 2.4. mRNA Construct Design and In Vitro Transcription

The mRNA vaccines were transcribed from plasmid templates in vitro. Briefly, the recombinant pVAX-HA-GRA35 plasmid was linearized using appropriate restriction endonucleases (Share-bio, Shanghai, China) and purified with a PCR product recovery kit (Share-bio, Shanghai, China) prior to in vitro transcription. GRA35 mRNA was subsequently synthesized using a T7 RNA polymerase-based in vitro transcription kit (Yeasen Biotechnology, Shanghai, China) according to the manufacturer’s instructions, the reaction mixture contained T7 RNA polymerase, NTP Mix (contain modified nucleotides), and a cap analog (ARCA) to achieve mRNA synthesis and co-transcriptional capping. In addition, the *E. coli* Poly(A) Polymerase (Beyotime, Shanghai, China) was added to the 3′ end of the mRNA according to the manufacturer’s instructions, thereby enhancing its stability and translational efficiency in vivo. After transcription, the reaction mixture was treated with DNase I to remove residual template DNA, and the synthesized mRNA was purified using a commercial RNA purification kit (Beyotime, Shanghai, China). The integrity and size of the purified GRA35 mRNA were assessed by agarose gel electrophoresis, and the mRNA concentration and purity were assessed using a spectrophotometer by measuring absorbance at 260 nm (A260) and calculating the A260/A280 ratio. The purified mRNA was aliquoted and stored at −80 °C until further use. The in vitro–transcribed (IVT) mRNA construct consisted of five structural elements to enhance stability and translational efficiency, including a 5′ cap structure, a 5′ untranslated region (UTR), the open reading frame (ORF) encoding GRA35, a 3′ untranslated region (UTR), and a poly(A) tail.

### 2.5. Formulation and Characterization of mRNA-LNP

Lipid nanoparticles (LNP) were composed of four different lipids including SM-102, 1,2-distearoyl-sn-glycero-3-phosphocholine (DSPC), cholesterol and 1,2-dimyristoyl-rac-glycero-3-methoxypolyethylene glycol 2000 (DMG-PEG2000) bought from Medchem Express (Shanghai, China) at lipid molar ratios of 50:10:38.5:1.5 respectively, dissolved in absolute ethanol. LNP formulation, mRNA diluted in an aqueous buffer was rapidly mixed with lipid components dissolved in ethanol at a defined ratio to allow spontaneous self-assembly of mRNA-LNPs. Specifically, the aqueous and ethanol phases were combined at a 3:1 volume ratio. The resulting mRNA-LNP suspension was incubated at room temperature and then buffer-exchanged into phosphate-buffered saline (PBS) for in vivo use and stored at −80 °C until use. Encapsulation efficiency was more than 90% for all mRNA-LNPs in this study determined by Ribogreen (Invitrogen, NY, USA). Particle size and zeta potential of LNPs were evaluated using dynamic light scattering (DLS) instrument from ZS-90 (Malvern, Malvern, UK) and BeNano 90 (Bettersize, Dandong, China). The mean hydrodynamic diameter of mRNA-LNPs was 80 nm with a polydispersity index below 0.2. Two or three batches from each mRNA-LNP formulations were used in these studies and no variability in vaccine efficacy was observed.

### 2.6. Expression of GRA35 In Vitro

An indirect immunofluorescence assay (IFA) was performed to evaluate the in vitro expression of GRA35. Briefly, HEK 293T cells were transfected with GRA35 using Lipofectamine 2000 reagent (Invitrogen, Carlsbad, CA, USA) according to the manufacturer’s protocol, as previously described [21]. At 48 h post-transfection, the cells were fixed in pre-chilled 100% acetone for 30 min. After washing with PBS containing 0.1% Triton X-100 (PBST), the cells were incubated with anti-HA tag polyclonal antibody (Proteintech Group Inc., Chicago, IL, USA), followed by fluorescein isothiocyanate (FITC)-conjugated goat anti-rabbit IgG (Proteintech Group Inc., Chicago, IL, USA). The stained cell monolayers were mounted with glycerin and observed under a Zeiss Axioplan fluorescence microscope (Carl Zeiss, Oberkochen, Germany). HEK 293T cells transfected with the empty pVAX1 vector served as the negative control.

### 2.7. Vaccine Immunization and Challenge Infection

Mice (C57BL/6 and BALB/c) were used in equal numbers and randomly assigned to experimental and control groups using a block randomization approach performed independently for each strain. No predefined exclusion criteria were applied, and all animals were included in the analysis unless they died prior to the experimental endpoint. Outcome assessments, including cyst counting and flow cytometry analysis, were performed in a blinded manner, with investigators unaware of group allocation during data acquisition and analysis (Figure S1).

Immunizations were administered twice at 4-week intervals (weeks 0 and 4). Experimental groups received either the pVAX-GRA35 DNA vaccine (100 µg), GRA35 mRNA-LNP vaccine (10 µg), or heterologous prime–boost regimens (DNA prime/mRNA boost or mRNA prime/DNA boost) via intramuscular injection in a total volume of 100 µL sterile PBS. Control groups received LNP, empty pVAX1 vector, or PBS alone (100 µL per mouse). The selected doses were based on previous studies [20,23]. Two weeks after the final immunization, mice were euthanized for immunological analyses. Splenocytes were aseptically isolated and used for flow cytometric analysis and cytotoxic T lymphocyte (CTL) assays (*n* = 5), lymphocyte proliferation assays (*n* = 5), and cytokine production measurements (*n* = 5), memory T cell analysis (*n* = 5) was performed at 8 weeks post-final immunization. These experiments were performed in three independent replicates.

For challenge experiments, mice in each group were further subdivided. Two weeks after the final immunization, ten mice per group were intraperitoneally challenged with 1 × 10^3^ tachyzoites of the highly virulent *T. gondii* RH strain, while another ten mice were orally infected with 100 tissue cysts of the *T. gondii* PRU strain. Survival was monitored daily, and time to death was recorded. To assess chronic infection, five mice per group were orally challenged with 10 cysts of the PRU strain at 14 days post-immunization. Thirty days after challenge, mice were euthanized, and brain cyst burdens were quantified. Briefly, brain tissues were harvested and individually homogenized in 3 mL PBS. Cysts were enumerated by microscopic examination of 1 µL aliquots from each homogenate, and counts were performed in triplicate to calculate the total cysts burden per brain.

### 2.8. Measurement of Humoral Response

Serum samples were collected from all immunized mice at 0, 2, 4, and 6 weeks post-immunization. Antigen-specific IgG and IgG subclasses (IgG1, IgG2a, IgG2b, and IgG3) were quantified using the SBA Clonotyping System-HRP Kit (SouthernBiotech, Birmingham, UK) according to the manufacturer’s instructions following previously described protocols [21]. Briefly, plates were coated with *T. gondii* lysate antigen (TLA), and serum samples were applied to antigen-coated plates, and bound antibodies were detected using HRP-conjugated subclass-specific secondary antibodies. The absorbance was measured at 450 nm using a microplate reader (BioTek EL×800, VT, USA). All samples were analyzed in triplicate.

### 2.9. Lymphocyte Proliferation Assay

Two weeks after the final immunization, five mice per group were euthanized, and splenocytes were aseptically isolated by mechanical dissociation through a wire mesh. Red blood cells were removed using RBC lysis buffer (Sigma, MA, USA). Splenocytes were seeded in triplicate at a density of 1 × 10^6^ cells per well in complete DMEM supplemented with 10% fetal bovine serum, 100 IU/mL penicillin, and 100 IU/mL streptomycin. Cells were stimulated with *Toxoplasma* lysate antigen (TLA; 10 µg/mL), concanavalin A (Con A; 5 µg/mL; Sigma, USA) as a positive control, or medium alone as a negative control, and incubated for 72 h at 37 °C in a humidified atmosphere containing 5% CO_2_. Cell proliferation was assessed using the MTT assay. Briefly, 10 µL of MTT solution (5 mg/mL; Sigma, USA) was added to each well and incubated for an additional 4 h. The absorbance was measured at 570 nm, and the stimulation index (SI) was calculated as follows: SI = OD_570_ (stimulated)/OD_570_ (unstimulated control).

### 2.10. Western Blotting Analysis

Expression of recombinant GRA35 protein, as well as GRA35 mRNA and DNA, was evaluated in HEK 293T cells. Cells were transfected with empty pVAX1 vector, GRA35 mRNA-LNP, or pVAX-GRA35 DNA. Protein expression was analyzed by Western blotting (WB), with GAPDH as an internal control. Detection of GRA35 was performed using anti-HA tag polyclonal antibody (dilution 1:1000) as the primary antibody. The detailed experimental procedures were carried out as described previously [24].

### 2.11. Flow Cytometry

Flow cytometry was used to determine the proportions of CD3^+^ T cells, CD4^+^ and CD8^+^ T-cell subsets, memory T-cell subsets (CD44^+^CD62L^+^ and CD44^+^CD62L^−^), and CD80^+^, CD86^+^, MHC I^+^, MHC II^+^ DCs in splenocytes from mice in different experimental groups, as previously described [21]. Splenocytes (1 × 10^6^ cells/mL) were incubated with fluorochrome-conjugated antibodies, including Live/Dead-ECD (Thermo Fisher Scientific, MA, USA), PE-CD3, BV421-CD4, PerCP-CD8, PE-CD80, BV605-CD86, APC-MHC-I, and FITC-MHC-II, BV421-CD44, APC-CD62L (BD, CA, USA), for 30 min at 4 °C in the dark. Cells were washed with PBS, resuspended in PBS, and acquired on a FACScan flow cytometer (BD, CA, USA) and analyzed using SYSTEM II software (V2.4) (BD, CA, USA). Each sample was tested in triplicate using splenocytes from five mice.

### 2.12. Cytokine Assays

Fourteen days after the final immunization, five mice per group were randomly selected and euthanized according to ethical guidelines. Serum samples were collected to measure the expression levels of IL-2, IL-4, IL-6, IL-10, IL-12, and IFN-γ using ELISA kits (Elabscience, Wuhan, China) according to the manufacturer’s instructions. During this period, spleens were aseptically removed to prepare single-cell suspensions. Splenocytes (5 × 10^6^ cells/well) were cultured in 24-well plates at 37 °C with 5% CO_2_ for 72 h. Supernatants were then collected for cytokine analysis using the same ELISA kits and protocols as described above [19].

### 2.13. Cytotoxic T Lymphocyte (CTL) Activity Assay

Splenocytes were isolated as described above, and cytotoxic T lymphocyte (CTL) activity was assessed using the CytoTox96^®^ Non-Radioactive Cytotoxicity Assay Kit (Promega, WI, USA) according to the manufacturer’s instructions. For the generation of effector cells, splenocytes were stimulated with recombinant murine IL-12 (100 U/mL; Selleck, TX, USA). Target cells consisted of Sp2/0 mouse cells transfected with the eukaryotic expression plasmid using Lipofectamine™ 2000 (Invitrogen, CA, USA) and cultured for 5 days prior to the assay. Effector and target cells were co-incubated at effector-to-target (E:T) ratios of 10:1, 20:1, 40:1, and 80:1 for 6 h. Cytotoxic activity was determined by measuring lactate dehydrogenase (LDH) release, and the percentage of specific lysis was calculated as follows: Specific lysis (%) = [(experimental release − effector spontaneous release − target spontaneous release)/(target maximum release − target spontaneous release)] × 100.

### 2.14. Statistical Analysis

All statistical analyses were performed using GraphPad Prism 9.5 and SPSS Statistics 17.0 (IBM, NY, USA). Differences among groups in the obtained data (including antibody responses, lymphocyte proliferation assays, and cytokine secretion) were assessed using one-way or two-way ANOVA. Survival outcomes were displayed as Kaplan–Meier curves and analyzed by the log-rank test. A *p*-value < 0.05 was considered to indicate statistical significance between groups.

## 3. Results

### 3.1. Confirmation and Characterization of Plasmid Constructs

**Figure 1:**
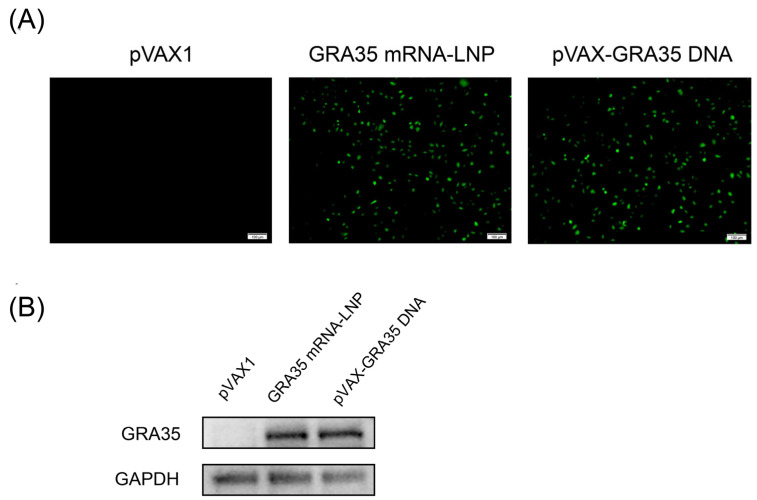
Detection of recombinant GRA35 protein expression along with mRNA and DNA expression in HEK 293T cells. HEK 293T cells were transfected with empty pVAX1, GRA35 mRNA-LNP, pVAX-GRA35 DNA. Cells transfected with empty pVAX1 served as negative controls. (**A**) The expression of GRA35 mRNA-LNP or pVAX-GRA35 DNA in HEK 293T cells by IFA. (**B**) Detection of GRA35 protein by Western blotting.

Bioinformatic analyses indicated that GRA35 contains multiple predicted B-cell epitopes and exhibits favorable MHC class II binding potential, supporting its suitability as a vaccine candidate (Figure S2 and Table S1). Specific green fluorescence was observed in HEK 293T cells transfected with GRA35 mRNA-LNP or pVAX-GRA35 DNA, confirming the presence of positive plasmids, whereas no fluorescence was detected in cells transfected with the empty pVAX1 vector (Figure 1A). Additionally, protein expression from GRA35 mRNA and DNA in vitro was detected by Western blotting (Figure 1B). The rabbit anti-GRA35 polyclonal antibody detected a single specific protein band in HEK 293T cells transfected with GRA35 mRNA-LNP or pVAX-GRA35 DNA, whereas no band was detected in untransfected HEK 293T cells. These results demonstrated that GRA35 could be successfully expressed from both GRA35 mRNA-LNP and pVAX-GRA35 DNA in eukaryotic cells.

### 3.2. Analysis of Humoral Immune Responses in Immunized Mice

The humoral immune response in immunized and control mice was evaluated by measuring IgG levels in serum samples collected at weeks 0, 2, 4, and 6 after immunization, and by detecting IgG1, IgG2a, IgG2b, and IgG3 isotype distribution at 2 weeks after the final injection, both using standard ELISA.

**Figure 2:**
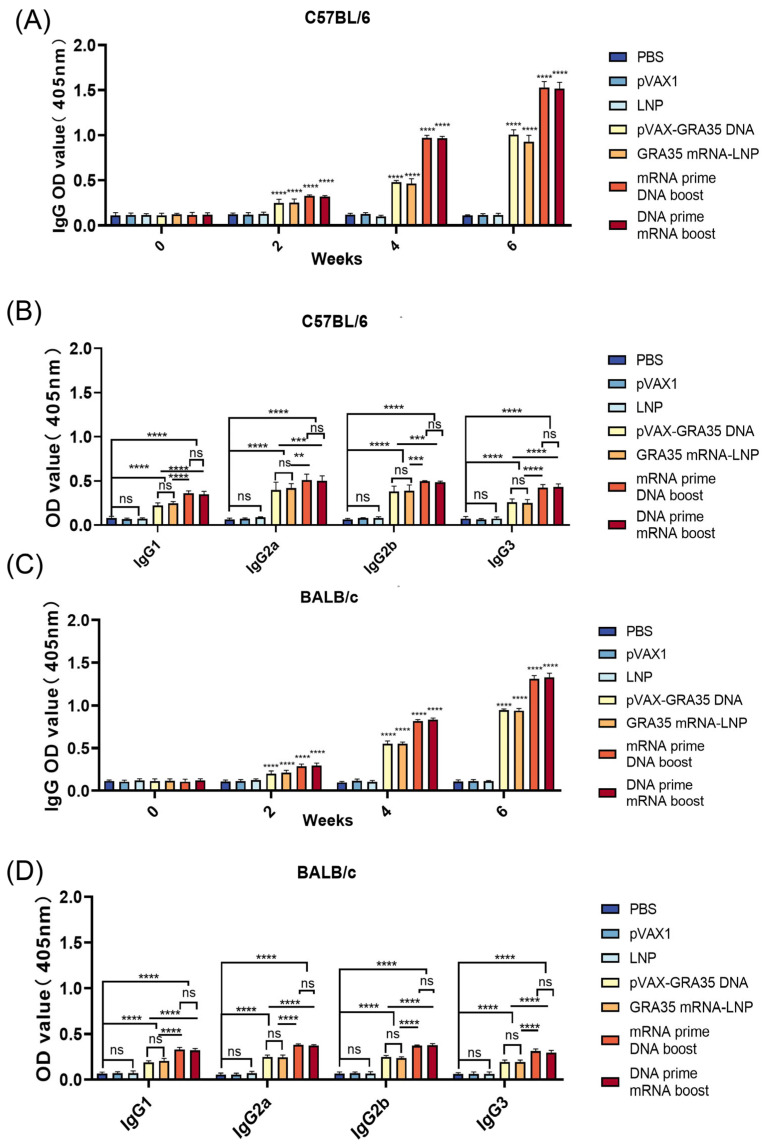
Detection of specific anti-*T. gondii* humoral immune responses induced by different types of vaccines (*n* = 5 mice per group). Determination of IgG antibodies in the sera of C57BL/6 mice (**A**) and BALB/c mice (**C**) at 0, 2, 4, and 6 weeks. Detection of IgG1, IgG2a, IgG2b, IgG3 antibodies in immunized C57BL/6 (**B**) and BALB/c mice (**D**) 2 weeks after the last immunization. Results were expressed as mean ± SD. Statistical analysis was performed using one-way or two-way ANOVA followed by multiple comparison tests. ** *p* < 0.01, *** *p* < 0.001, **** *p* < 0.0001; ns, not significant.

As shown in Figure 2A, in C57BL/6 mice, serum from all GRA35-encoding vaccine groups exhibited significantly higher IgG levels compared with the control groups. Heterologous mRNA and DNA prime–boost regimens elicited the strongest antibody responses, with no statistically significant difference detected between them. No significant increase in antibody titers was observed in the three control groups. Similar kinetics were observed in BALB/c mice (Figure 2C). As shown in Figure 2B,D, the GRA35-encoding vaccine groups exhibited significantly increased IgG1, IgG2a, IgG2b, and IgG3 endpoint titers compared with control groups in both C57BL/6 and BALB/c mice. The heterologous prime–boost regimens consistently induced the highest titers across all groups in both mouse strains, although no significant difference was detected between the GRA35 mRNA-LNP prime/pVAX-GRA35 DNA boost and pVAX-GRA35 DNA prime/GRA35 mRNA-LNP boost groups.

These results indicate that the heterologous prime–boost strategies effectively enhance humoral immunity and induce a mixed Th1/Th2-type humoral immune response.

### 3.3. Evaluation of T Cell-Mediated Immune Responses

**Figure 3:**
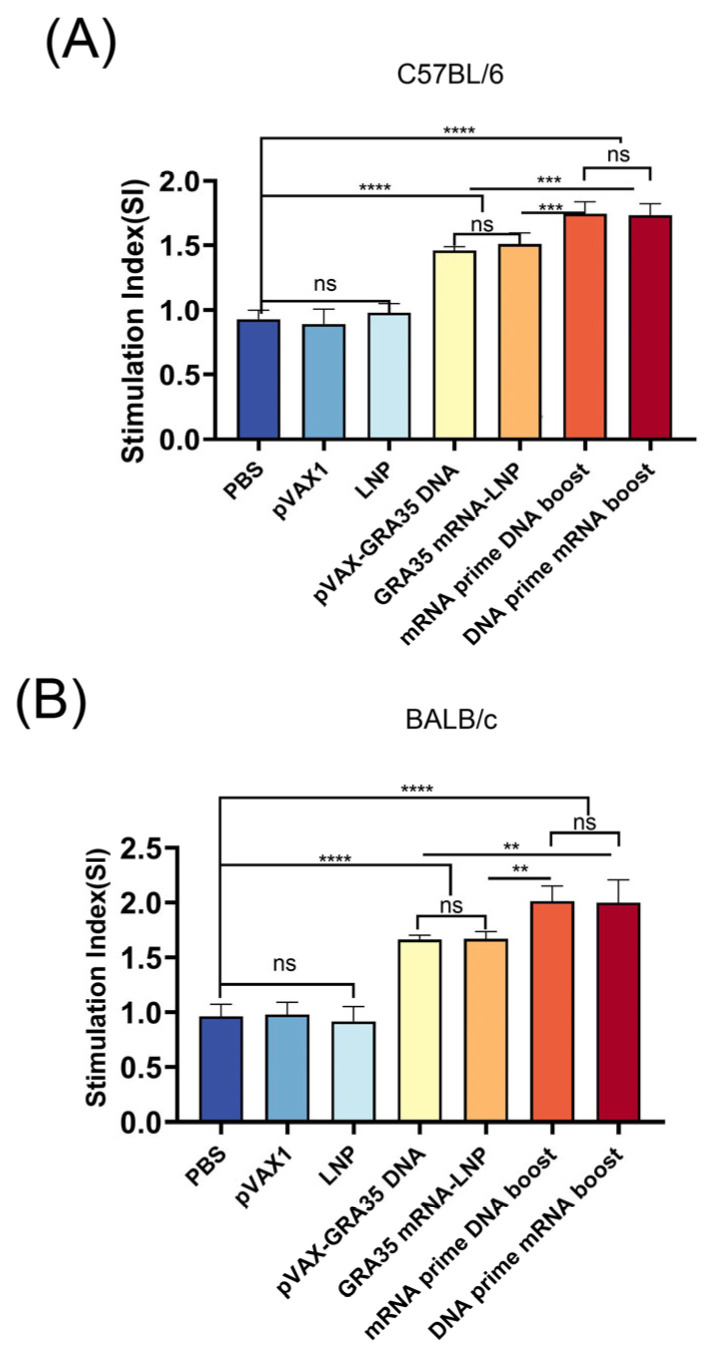
Proliferation of splenocytes from immunized and control mice (*n* = 5 mice per group). (**A**) Lymphocyte proliferation stimulation index in C57BL/6 mice. (**B**) Lymphocyte proliferation stimulation index in BALB/c mice. Results were expressed as mean ± SD. Statistical analysis was performed using one-way ANOVA followed by multiple comparison test. ** *p* < 0.01, *** *p* < 0.001, **** *p* < 0.0001; ns, not significant.

The MTT assay was used to assess splenocyte proliferative responses to TLA and Con A stimulation, performed two weeks after the final immunization. As shown in Figure 3, following stimulation with TLA, Con A, C57BL/6 and BALB/c mice immunized with GRA35-encoding vaccines displayed significantly enhanced lymphoproliferative responses, as reflected by higher stimulation indices (SI) measured at OD450nm relative to the PBS, LNP, and pVAX1 control groups. Moreover, the heterologous prime–boost strategies significantly elevated the stimulation index (SI) compared with single-dose mRNA or DNA vaccination. However, no significant difference was observed among the vehicle control groups.

**Figure 4:**
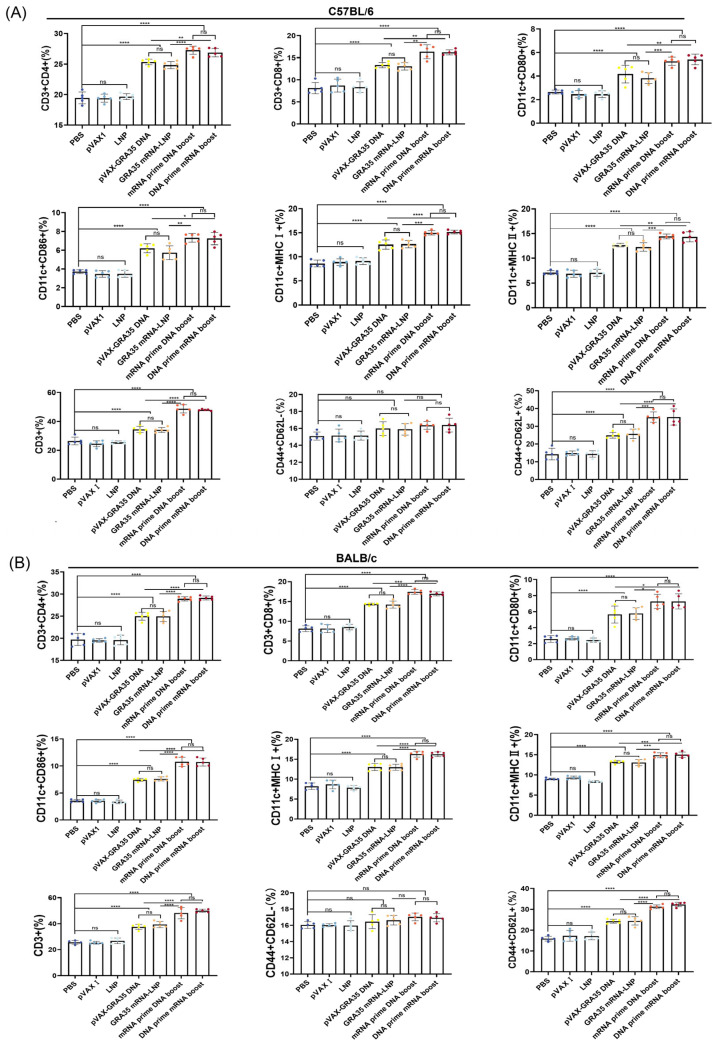
Flow cytometric analysis of cellular immune responses in immunized mice. The percentages of CD3^+^, CD4^+^ and CD8^+^ T cells, and CD80^+^, CD86^+^, MHC I^+^, and MHC II^+^ dendritic cells, CD44^+^CD62L^−^, CD44^+^ and CD62L^+^ in immunized and control mice in C57BL/6 mice (**A**) and BALB/c mice (**B**) (*n* = 5 mice per group). Results were expressed as mean ± SD. Statistical analysis was performed using one-way ANOVA followed by multiple comparison test. * *p* < 0.05, ** *p* < 0.01, *** *p* < 0.001, **** *p* < 0.0001; ns, not significant.

In addition, flow cytometry was employed to further investigate cellular immune responses by analyzing the percentages of CD8^+^ and CD4^+^ T cells and CD80^+^, CD86^+^, MHC I^+^, and MHC II^+^ DCs in the spleens of C57BL/6 and BALB/c mice from each group. As shown in Figure 4, the percentages of T cell and DC subsets in the spleens of all mice immunized with GRA35-encoding vaccines were significantly higher than those in the control groups, and consistent trends were observed in both C57BL/6 and BALB/c mouse strains. The heterologous prime–boost regimens elicited a greater proportion of CD8^+^ and CD4^+^ T cells, as well as CD80^+^, CD86^+^, MHC I^+^, MHC II^+^ DCs, CD44^+^ and CD62L^+^ compared with the single-dose mRNA and DNA groups, highlighting the heterologous prime–boost regimens significantly enhanced the cellular immune response.

### 3.4. Assessment of Cytokine Levels and Cytotoxic T Cell Function

**Figure 5:**
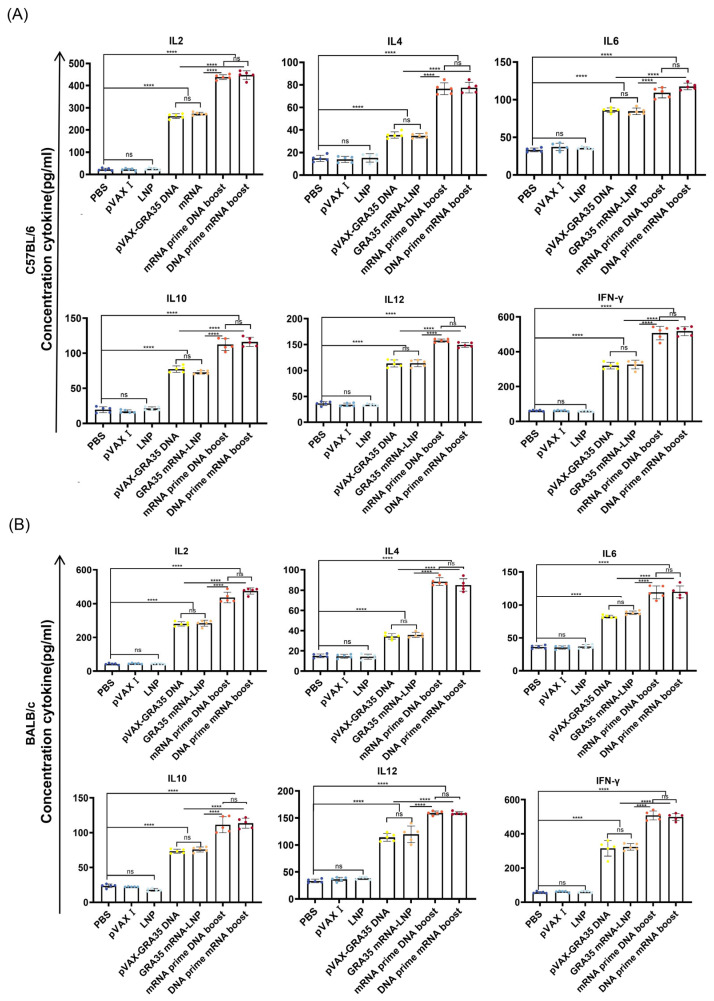
Cytokine production by serum from immunized C57BL/6 (**A**) and BALB/c (**B**) mice following in vitro stimulation with *T. gondii* lysate antigen (TLA) (*n* = 5 mice per group). Levels of IL6, Th1-associated cytokines (IFN-γ, IL-2, and IL-12) and Th2-associated cytokines (IL-4 and IL-10) were quantified by ELISA. Results were expressed as mean ± SD. Statistical analysis was performed using one-way ANOVA followed by multiple comparison test. **** *p* < 0.0001; ns, not significant.

Two weeks after the final immunization, splenocytes and serum samples were harvested from C57BL/6 and BALB/c mice. Splenocyte cytokine levels were assessed by ELISA following stimulation with TLA, whereas serum cytokine levels were measured by the same method without stimulation. As shown in Figure 5, the levels of IL6, Th1-associated cytokines (IFN-γ, IL-2, and IL-12) and Th2-associated cytokines (IL-4 and IL-10) were significantly elevated in mice immunized with the GRA35-encoding vaccines compared with those immunized with vehicle control groups. C57BL/6 mice receiving heterologous mRNA and DNA prime–boost regimens exhibited the highest cytokine levels among all groups, whereas the three control groups showed no significant differences among themselves (Figure 5A). Similar immune responses were observed in BALB/c mice (Figure 5B) and splenocyte culture supernatants (Figure S3).

**Figure 6:**
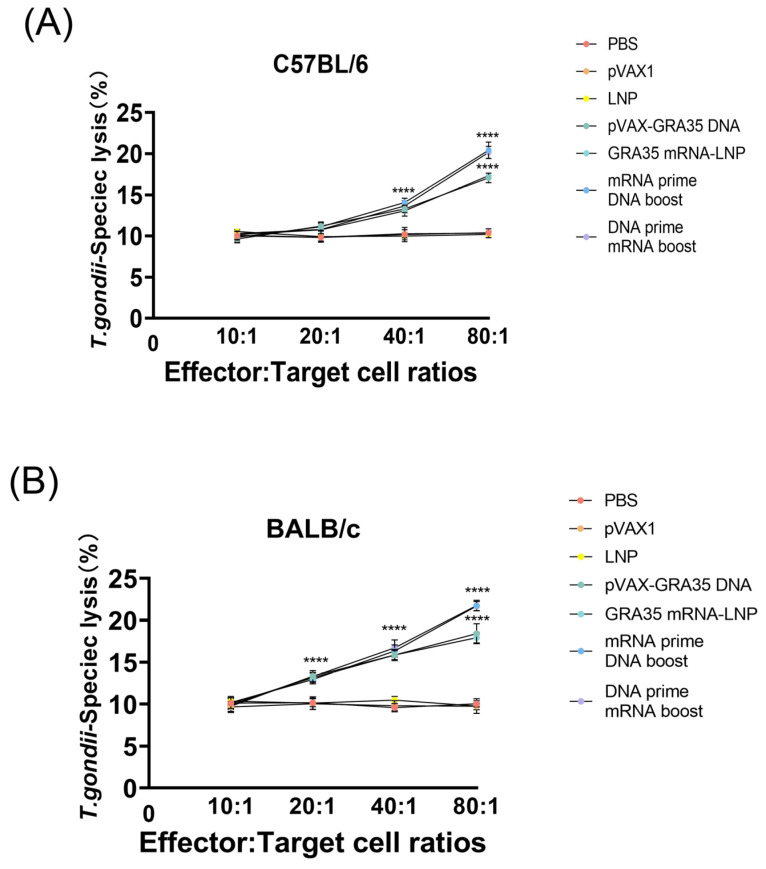
Cytotoxic T lymphocyte activities of splenocytes from differentially immunized mice (*n* = 5 mice per group). CTL activity was measured at various effector-to-target (E:T) ratios ranging from 10:1 to 80:1 *T. gondii*-specific lysis in C57BL/6 mice (**A**) and BALB/c mice (**B**). Results were expressed as mean ± SD. Statistical analysis was performed using two-way ANOVA followed by multiple comparisons test. **** *p* < 0.0001.

The CTL activity of splenocytes derived from all GRA35-encoding vaccine-immunized mice increased progressively with the effector-to-target cell ratio, reaching a peak at an 80:1 ratio (Figure 6). Moreover, both C57BL/6 and BALB/c mice receiving the heterologous prime–boost regimen exhibited the highest CTL activity, with no significant difference between them. In contrast, the three control groups showed no significant differences among themselves.

### 3.5. Evaluation of the Protective Efficacy in Immunized Mice

**Figure 7:**
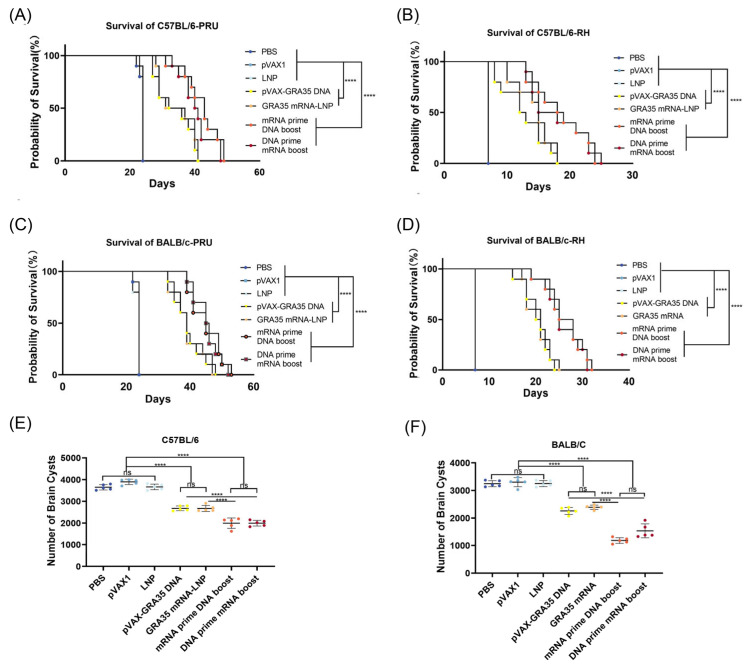
The survival rate of immunized mice challenged with the RH or PRU strain (*n* = 10 mice per group). The vaccine protected immunized C57BL/6 mice against challenge with the PRU (**A**) or the RH (**B**) strain. Protection induced by active immunization with vaccine against PRU (**C**) or RH (**D**) strain challenge in immunized BALB/c mice. Statistical analysis was performed by log-rank tests. Protection against chronic toxoplasmosis in immunized mice at 2 weeks post-boost (*n* = 5 mice per group). Brain cyst counts in immunized C57BL/6 mice (**E**) or BALB/c mice (**F**). Data were analyzed by one-way ANOVA with multiple comparisons. **** *p* < 0.0001; ns, not significant.

Survival of C57BL/6 and BALB/c mice was monitored after challenge with 100 cysts of the PRU strain or 10^3^ tachyzoites of the RH strain to assess the protective efficacy of the immunization protocol. As shown in Figure 7, mice immunized with GRA35-encoding vaccines exhibited significantly longer survival times than control groups in both C57BL/6 and BALB/c mice (*p* < 0.0001). Immunization with heterologous mRNA and DNA prime–boost regimens resulted in the longest survival time following RH and PRU challenge. Single-dose mRNA and DNA vaccines showed intermediate and modest protection, and all control mice succumbed to infection within 25 days (PRU) or 10 days (RH).

To assess the protective efficacy against chronic infection by the *T. gondii* PRU strain, the number of cysts in the brains of immunized experimental mice was examined. As shown in Figure 7E,F, significantly fewer brain cysts were observed in the GRA35-encoding vaccine groups compared with the control groups in C57BL/6 mice and BALB/c mice. In addition, GRA35 DNA/mRNA prime–boost regimens resulted in a highly significant reduction in brain cyst numbers (*p* < 0.0001) compared with the control group. In contrast, high cyst burdens persisted in all control groups, with no significant reduction observed. These results demonstrate that GRA35-encoding vaccines, particularly heterologous mRNA and DNA prime–boost regimens, confer robust protection against both chronic and acute *T. gondii* infections.

## 4. Discussion

Despite extensive efforts in *T. gondii* vaccine development, most studies have pre-dominantly focused on antigen discovery and selection, while the role of vaccine platform and immunization strategy in shaping protective immunity remains insufficiently defined. In particular, whether distinct nucleic acid platforms can be rationally combined to optimize immune responses against intracellular parasites has not been systematically investigated. The study addressed this gap by using GRA35 as a model antigen to directly compare DNA and mRNA vaccine platforms and to evaluate homologous versus heterologous prime–boost strategies. Our results demonstrate that heterologous DNA–mRNA immunization significantly enhances both the magnitude and quality of protective immunity against acute and chronic *T. gondii* infection. Importantly, our findings suggest that vaccine efficacy against intracellular pathogens is not solely determined by antigen selection, but is instead critically influenced by the integration of complementary antigen expression platforms. This work therefore shifts the focus from antigen-centered design toward platform-driven optimization of immune responses.

Over the past two decades, DNA vaccine development against *T. gondii* has largely focused on antigen selection, including surface proteins, dense granule proteins, and rhoptry effectors. Early studies targeting SAG1 demonstrated induction of Th1-associated cytokines and partial protection in murine models, although overall efficacy was limited [25]. Subsequent investigations of dense granule antigens such as GRA7 [26] and GRA24 [5] reported enhanced cellular immune responses and reductions in brain cyst burden, while rhoptry proteins such as ROP5 were evaluated due to their roles in parasite virulence and host immune modulation [27]. Nevertheless, monovalent DNA vaccines based on individual antigens generally conferred incomplete protection, particularly against highly virulent strains. Multi-antigen combinations have been explored to broaden immune coverage and improve efficacy [28], though these approaches increase construct complexity and may introduce challenges related to antigen balance and expression. Rather than expanding antigen repertoire, the present study evaluated whether heterologous DNA-mRNA prime–boost immunization could enhance immune responses using a single dense granule antigen, GRA35. The observed increases in T-cell activation, cytokine production, and reduction in cyst burden suggest that optimization of vaccine platform design and antigen delivery dynamics may contribute to improved protective efficacy alongside antigen selection in the development of vaccines against *T. gondii*.

Heterologous prime–boost strategies have been successfully applied in vaccines against complex pathogens such as HIV/AIDS [29], malaria [30], and tuberculosis [31], primarily to overcome anti-vector immunity and enhance immunogenicity. Unlike viral-vector-based regimens, some studies have reported that DNA and mRNA vaccines share a common principle of intracellular antigen synthesis, although they engage partially distinct innate sensing pathways [32,33]. DNA vaccines are generally thought to engage cytosolic DNA-sensing pathways, such as cGAS–STING, whereas mRNA vaccines may activate endosomal and cytosolic RNA sensors including TLR7/8 and RIG-I-like receptors [34,35,36]. Although these pathways were not directly investigated in the present study, they may provide a potential explanation for the immune responses observed. The convergence of these pathways on dendritic cell maturation and type I interferon signaling may partially explain the comparable immunogenicity observed regardless of immunization order in our study. These findings suggest that, when two platforms ultimately induce intracellular antigen production and efficient MHC class I cross-presentation, the sequence of administration exerts limited influence on the magnitude of adaptive immune output.

Importantly, the two platforms differ in antigen expression kinetics. DNA vaccines typically mediate relatively sustained but moderate antigen expression, potentially favoring prolonged antigen presentation and memory T-cell programming [37]. In contrast, mRNA vaccines generate rapid, high-level yet transient antigen expression, which may preferentially drive strong initial effector responses and germinal center formation [38]. This study speculates that the integration of these complementary kinetic profiles contributes to the enhanced CD8^+^ cytotoxic T lymphocyte (CTL) activity and accelerated antibody production observed in the heterologous groups [39,40]. Rather than acting redundantly, the platforms may cooperatively shape both the amplitude and temporal dynamics of antigen exposure and optimize immune priming and recall responses. Collectively, our findings support a model in which distinct nucleic acid vaccine platforms provide complementary immunological inputs rather than redundant functions. DNA vaccines may contribute to sustained antigen availability and prolonged immune priming, whereas mRNA vaccines may drive rapid and robust effector responses. The integration of these platforms through heterologous prime–boost immunization may therefore enable coordinated control over both the magnitude and temporal dynamics of antigen exposure, ultimately enhancing the quality of adaptive immunity.

T-cell-mediated immunity is central to resistance against *T. gondii*. The marked expansion of CD3^+^ CD4^+^ and CD8^+^ T-cell populations, together with enhanced parasite-specific cytolytic activity, indicates that heterologous vaccination effectively promotes functional effector differentiation [41]. Given that control of intracellular parasites depends on efficient cross-presentation and IFN-γ-dependent macrophage activation [42], the observed upregulation of CD80^+^, CD86^+^, MHC-I^+^, and MHC-II^+^ on dendritic cells suggests improved antigen presentation capacity and reduced activation thresholds for naïve T cells [43]. The simultaneous engagement of DNA- and RNA-sensing pathways may contribute to enhanced dendritic cell maturation signals, thereby strengthening CD8^+^ T-cell priming and sustaining Th1 polarization. The observed increase in CD44^+^CD62L^+^ central memory T cells may indicate the formation of long-lived immunological memory, potentially allowing rapid proliferation and strong secondary responses following re-exposure to *T. gondii*. This coordinated activation may underlie the superior reduction in brain cyst burden and prolonged survival following challenge with both RH and PRU strains.

Protective immunity against toxoplasmosis is classically characterized by Th1 dominance, particularly IFN-γ and IL-12 production [44]. The data demonstrate robust induction of these cytokines in the heterologous groups, while also detecting elevated IL-4 and IL-10 levels [45]. Rather than indicating immune deviation, this mixed cytokine profile likely reflects a balanced response that combines strong parasite control with regulation of excessive inflammation. The simultaneous enhancement of IgG subclasses associated with both Th1- and Th2-type responses further supports the notion that heterologous nucleic acid vaccination does not merely amplify one arm of immunity but promotes integrated immune programming [46,47]. Such balanced responses may be particularly important in preventing immunopathology during chronic infection. Furthermore, the robust induction of IL-6 reflects effective vaccine-triggered inflammatory and Th17-associated immune activation, which contributes to enhanced cellular protection against *T. gondii* infection. The selection of GRA35 as the vaccine antigen was supported by bioinformatic analyses revealing favorable antigenic indices, hydrophilicity, and predicted B- and T-cell epitopes [19]. Dense granule proteins play critical roles in host–parasite interactions, and targeting secreted effectors may enhance immune recognition during intracellular stages [18]. In this study, two murine models with distinct genetic backgrounds (BALB/c and C57BL/6) were employed to evaluate vaccine candidates, demonstrating protective efficacy in both strains. Our findings further suggest that this regimen is robust across different immunogenetic backgrounds. GRA35-encoding vaccines significantly extended survival duration and diminished cerebral tissue cyst burdens in immunized mice relative to control groups. The marked reduction in cerebral cyst burdens observed in both BALB/c and C57BL/6 mice, coupled with the significantly more pronounced survival prolongation demonstrated in BALB/c mice relative to C57BL/6 mice. These findings suggest that the heterologous vaccination strategy is effective across different immunogenetic backgrounds, supporting its potential robustness and broader applicability. Importantly, the ability to reduce cyst burden in the brain highlights the potential of nucleic acid–based heterologous vaccination to influence chronic infection, which remains a major obstacle in toxoplasmosis control [22]. These findings indicated that the heterologous mRNA and DNA prime–boost regimens confer effective protection against both acute lethal infection and chronic cyst formation, a critical requirement for next-generation *T. gondii* vaccines.

In this investigation, heterologous mRNA and DNA prime–boost regimens targeting GRA35 amplified humoral and cellular immune reactions, manifested by elevated lymphocyte proliferation, augmented IgG and subclass production and potentiated Th1-biased cytokine secretion alongside CD8^+^ cytotoxic T cell responses. Consequently, vaccinated mice exhibited considerable protection against acute and chronic *T. gondii* challenges. Despite these encouraging results, several limitations warrant consideration. This study did not assess long-term memory B-cell phenotypes, tissue-resident lymphocyte populations, histopathology or the durability of protection beyond the acute observation period. Furthermore, the precise innate signaling cascades responsible for the observed synergy were not dissected using pathway-deficient models. The use of only one sex of mice is also acknowledged as a limitation, as is the absence of an in vivo antigen expression assay. To address these limitations, future studies will include both sexes to evaluate potential sex-specific differences in vaccine efficacy and will incorporate an in vivo antigen expression assay and to further strengthen the conclusions. Additionally, future studies with expanded sample sizes, integrating transcriptomic profiling of dendritic cells, neutralization assays and additional characterization of memory subsets will be essential to define the mechanistic basis of cross-platform immune programming. Beyond toxoplasmosis, these findings may have broader implications for vaccine design against intracellular pathogens, where effective immunity requires coordinated cellular responses and precise regulation of antigen presentation dynamics.

## 5. Conclusions

Our study establishes heterologous mRNA and DNA prime–boost regimens targeting GRA35 that induce coordinated dendritic cell maturation, potent CTL responses, central memory T cells, balanced Th1-skewed cytokine production, and broad antibody immunity. The comparable efficacy observed irrespective of immunization order suggests that intracellular antigen synthesis-based platforms may converge on shared immunological pathways to achieve synergistic protection. This work provides a conceptual framework for nucleic acid vaccine integration against *T. gondii* and potentially other intracellular parasites, supporting the broader application of cross-platform prime–boost strategies in anti-parasitic vaccine development.

## Data Availability

The original contributions presented in this study are included in the article/Supplementary Material. Further inquiries can be directed to the corresponding authors.

## Supplementary Materials

The following supporting information can be downloaded at https://www.mdpi.com/article/10.3390/microorganisms14051000/s1: Figure S1: A clear schematic experimental timeline diagram; Figure S2: The plot of the DNASTAR predicted hydrophilicity, flexible regions, antigenic index, and surface probability of the GRA35 compared with those of SAG1; Figure S3: Splenocyte culture supernatants’ cytokine analysis; Figure S4: A full gating strategy; Figure S5: Original blot images. Table S1: IC50 values for GRA35 and SAG1 binding to MHC class II molecules, obtained using IEDB ^a^.

## Abbreviations

The following abbreviations are used in this manuscript:

CTL	Cytotoxic T lymphocyte
GRAs	Dense granule proteins
TLA	*Toxoplasma* lysate antigen
FBS	fetal bovine serum
IFA	indirect immunofluorescence assay
FITC	fluorescein isothiocyanate
RT-PCR	reverse transcription polymerase chain reaction
IVT	in vitro-transcribed
ORF	the open reading frame
LNP	Lipid nanoparticle
PBS	phosphate-buffered saline
DMEM	Dulbecco’s modified Eagle’s medium
Con A	concanavalin A
WB	Western blotting
SI	stimulation index
