# Supplementary material for: Heterologous Prime–Boost Vaccination with GRA35-Encoding DNA and mRNA Vaccines Enhances Protective Immunity Against Toxoplasma gondii Infection in Mouse Models
Source: Microorganisms. 2026 Apr 29;14(5):1000. doi: 10.3390/microorganisms14051000 (PMC13209544; doi:10.3390/microorganisms14051000)
Supplement: Supplementary file 1 [file microorganisms-14-01000-s001.zip › microorganisms-4203405-supplementary.pdf]

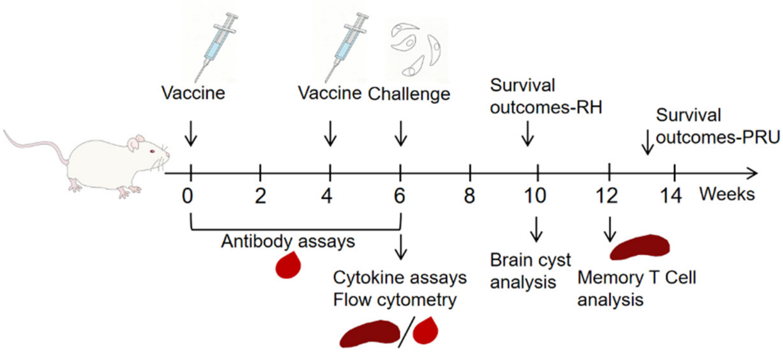

Figure S1. A clear schematic experimental timeline diagram.

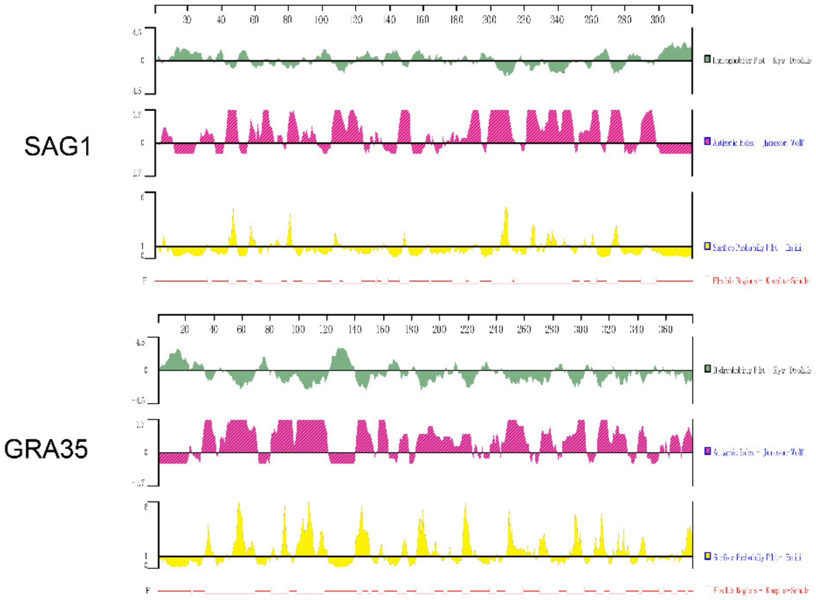

Figure S2. The plot of the DNASTAR predicted hydrophilicity, flexible regions, antigenic index, and surface probability of the GRA35 compared with those of SAG1.

Table S1 IC50 values for GRA35 and SAG1 binding to MHC class II molecules, obtained using IEDB [a]

MHC II alleles [b]	Start-stop [c]		Percentile rank [d]	
	SAG1	GRA35	SAG1	GRA35
H2-IAb	60–74	24–38	3.8	1.1
H2-IAd	223–237	48–62	2.3	0.1

H2-IEd	222–236	176–190	2.4	0.41
HLA-DRB 1*01:01	279–293	222–236	3.5	0.14

a. The immune epitope database (http://tools.immuneepitope.org/mhcii). b. H2-IAb, H2-IAd, and H2-IEd alleles are mouse MHC class II molecules; the HLA-DRB1*01:01 allele is a human MHC class II molecule. c. 15 amino acids were chosen for analysis. d. Low percentile indicates high level binding according to the software instructions.

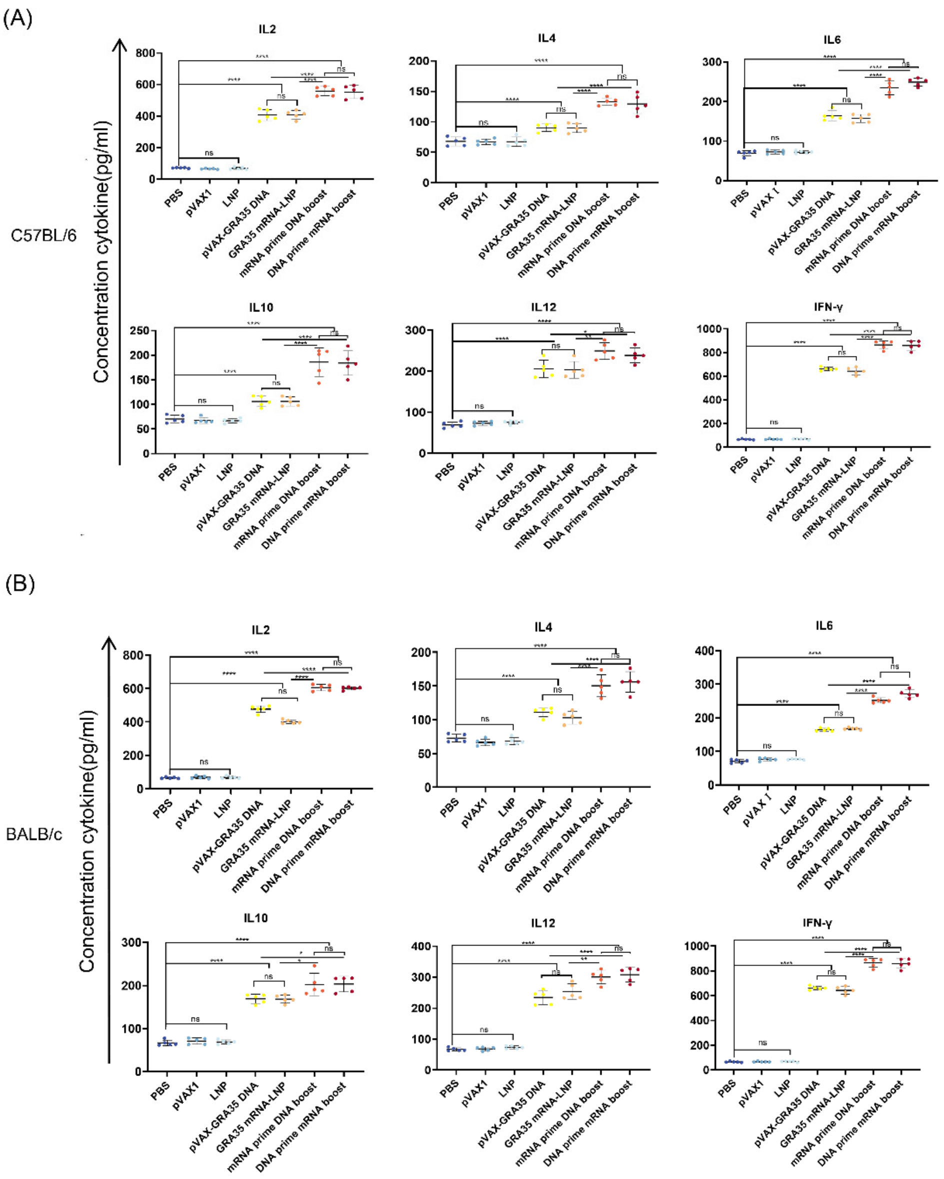

Figure S3. Splenocyte culture supernatants' cytokine analysis.

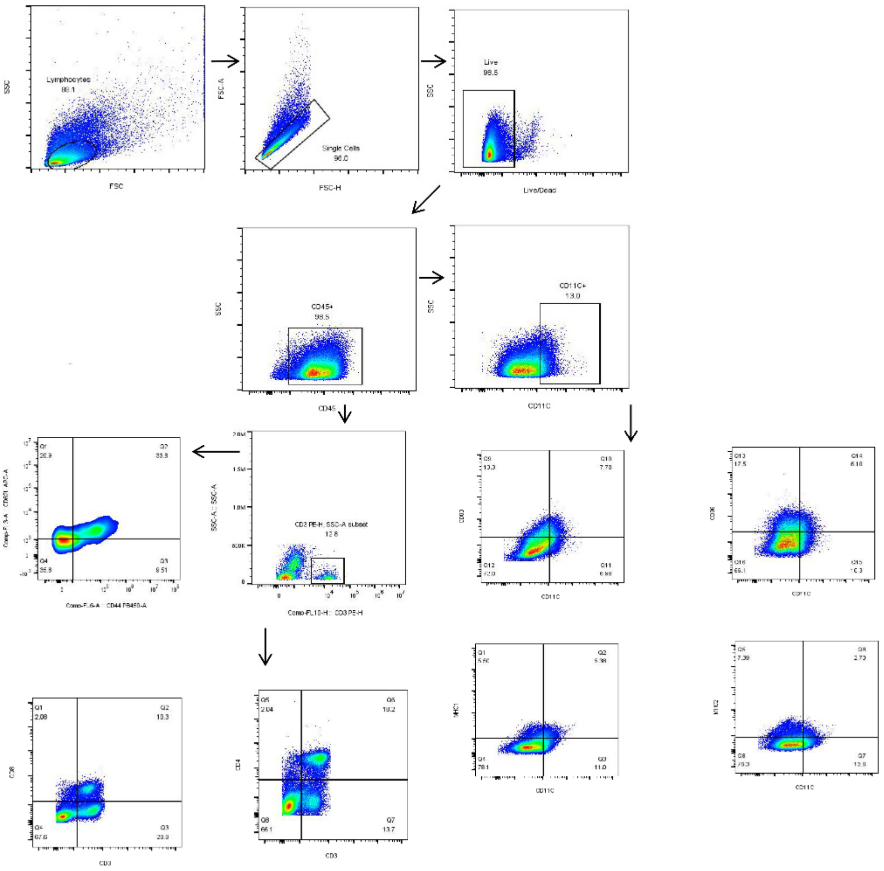

Figure S4. A full gating strategy.

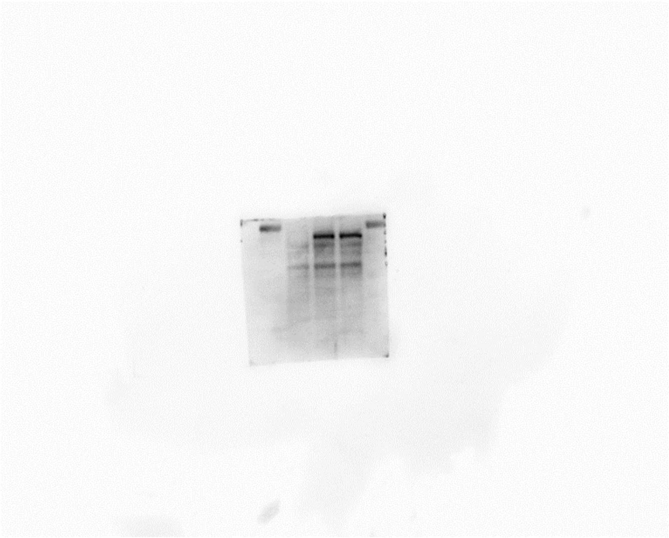

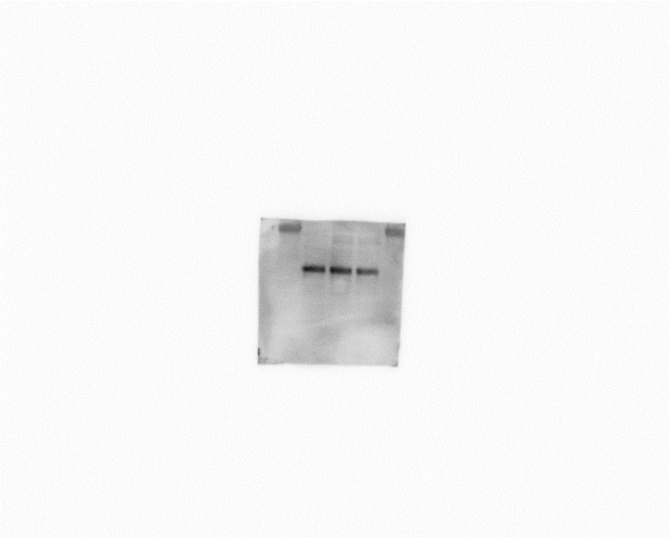

Figure S5. Original blot images.
